# Clinical characteristics and aetiology of uveitis in a viral haemorrhagic fever zone

**DOI:** 10.1038/s41433-024-03009-0

**Published:** 2024-05-15

**Authors:** Shiama Balendra, Lloyd Harrison-Williams, Jalikatu Mustapha, Zikan Koroma, Alicious Kamara, Bangi Saradugu, Osman Conteh, Theophilus Kanu, Santigie Kamara, Sheku Alhaji Koroma, Matthew Vandy, Laura Ward, Huachun Wang, Tolulope Fashina, Jessica Shantha, Steven Yeh, Alasdair Kennedy

**Affiliations:** 1 UCL-University College London, London, United Kingdom; 2 King’s College Global Health Partnership, King’s College London, London, United Kingdom; 3 University of Nebraska Medical Center, Omaha, NE 68198 USA; 4 Sight and Sound, Great Ormond Street Hospital, London, United Kingdom; 5 Jui Government Hospital, Freetown, Sierra Leone; 6 Connaught Hospital, Freetown, Sierra Leone; 7 Emory University, Atlanta, GA USA; 8 University of California San Francisco/Proctor Foundation, San Francisco, CA USA; 9 Moorfields Eye Hospital NHS Foundation Trust, London, EC1V 9EL UK

**Keywords:** Uveal diseases, Epidemiology, Inflammation, Infection, Inflammatory diseases

## Abstract

**Background/Objectives:**

Studies on uveitis in Sierra Leone were conducted prior to the Ebola Virus Disease epidemic of 2013–16, which was associated with uveitis in 20% of survivors. They did not include imaging or investigation of tuberculosis and used laboratory services outside the country. We performed a cross-sectional study on patients presenting with uveitis to establish their clinical characteristics and identify the impact of in-country laboratory diagnoses.

**Methods:**

We invited uveitis cases presenting to Eye Clinics in Sierra Leone from March to September 2022 to participate in the study. They underwent a diagnostic work-up, including fundus and ocular coherence tomography imaging. Active uveitis cases underwent further investigations including serology and immunological tests for syphilis, tuberculosis, herpetic viruses and HIV and chest radiographs.

**Results:**

We recruited 128 patients. The median age was 34 (IQR 19) years and there was an equal gender split. Panuveitis was the predominant anatomical uveitis type (*n* = 51, 40%), followed by posterior uveitis (*n* = 36, 28%). Bilateral disease affected 40 patients (31%). Active uveitis was identified in 75 (59%) cases. ICD 11 definition of blindness with VA < 3/60 occurred in 55 (33%) uveitis eyes. Aetiology of uveitis from clinical and laboratory assessment demonstrated that most cases were of undifferentiated aetiology (*n* = 66, 52%), followed by toxoplasmosis (*n* = 46, 36%). Trauma contributed to eight (6%) cases, syphilis to 5 (4%) cases and Ebola to 2 (2%).

**Conclusions:**

Uveitis was associated with high levels of visual impairment. Posterior and panuveitis contributed to the highest proportion of uveitis cases. Laboratory studies helped differentiate syphilis as a significant aetiology of uveitis.

## Introduction

Uveitis is intraocular inflammation originating from the uveal tract and adjacent structures. Its prevalence varies globally. In the USA, a prevalence of 115.3 cases per 100,000 of population has been reported [1]. In India, this was estimated at 714 per 100,000 [2]. Aetiology of uveitis, from studies in tertiary uveitis clinics, also varies globally. In the UK, Fuchs heterochromic uveitis (11.5%), sarcoid (9.7%), idiopathic uveitis (14.9%) and toxoplasmosis (6.9%) were the most common known diagnoses [3]. In the USA these were idiopathic uveitis (34.9%), seronegative spondyloarthropathies (10.4%) and sarcoidosis (9.6%) [4]. In China, idiopathic anterior uveitis accounted for 27% of anterior uveitis, and Behcet disease (6.5%) and Vogt-Koyanagi-Harada syndrome (15%) represented the most panuveitis cases [5]. In India, uveitis was caused by tuberculosis in 14.5%, toxoplasmosis in 11.7% and serpiginous choroidopathy in 14.6% [6].

West Africa has unique epidemiological characteristics with many endemic infectious diseases, making it challenging to extrapolate results from other areas. Studies of uveitis aetiology in West Africa are however limited. In Nigeria, anatomical subtypes of uveitis patients have been reported with the majority of posterior uveitis being of toxoplasmic origin [7]. The role of non-infectious autoimmune-disease related uveitis in Nigeria has also been highlighted [8]. In Benin, 85.7% of 489 patients with uveitis were reported to be idiopathic [9].

What is apparent is that uveitis is a significant cause of ocular morbidity in Sierra Leone. In 1992, Ronday published a hospital-based retrospective study. Uveitis was the second leading cause of blindness [10]. In another study by Ronday in 1996, infection accounted for over 50% of cases of uveitis with Toxoplasma Gondii and Treponema Pallidum, causing 43 and 20% of infective cases, respectively [10]. Sierra Leone has a population of 8.3 million and based on the afore mentioned prevalence studies from the USA and India, could have between 9000 and 55,000 cases of uveitis [1, 2, 11]. In reality, this figure may be even higher considering the endemic infectious diseases in-country. This represents a significant burden from a condition which affects people throughout their life course. Sight-threatening complications such as band keratopathy, cataract, macula oedema and glaucoma, have a major impact on quality of life and blindness also has socioeconomic costs [12].

Between 2013 and 2016, the Ebola Virus Disease (EVD) epidemic of West Africa affected 28,600 individuals [13]. The PREVAIL study in Liberia reported that 26% of Ebola Virus Disease (EVD) survivors and 12% of control patients show evidence of uveitis, a staggering proportion of individuals with no direct exposure to Ebola infection [14]. Uveitis occurs in approximately 20% of EVD survivors, raising questions regarding its impact on the aetiologic landscape of uveitis [15].

Although uveitis prevalence data is not available in Sierra Leone, data on other endemic infectious diseases, which may be associated with uveitis and have been reported by the Institute for Health Metrics and Evaluation as part of The Global Burden of Disease Study, may shed light on the scale of the problem [16]. In 2017, the prevalence of tuberculosis was 29%, syphilis was 1% and onchocerciasis (‘River Blindness’) was 5%. There was no estimation for toxoplasmosis or viruses capable of causing uveitis. HIV, associated with increased risk of toxoplasma or cytomegaloviral uveitis, was confirmed in 17.8% [17]. Sarcoidosis was reported in 0.03%.

The aim of this case series is to reveal the pattern of clinical phenotypes, sequelae, and disease associations of uveitis amongst patients presenting to the Eye Departments in Freetown, Sierra Leone. This study presents an opportunity to better understand uveitis in an understudied West African population who have been exposed to recent emergent public health threats including Ebola and Lassa fever.

## Methods

### Study type and patient recruitment

A prospective cross-sectional study was conducted between March to September 2022. With coordination from the National Eye Health Program of the Ministry of Health and Sanitation, patients were recruited from three study sites: Jui Hospital, Lowell and Ruth Gess Eye Hospital and Connaught Government Hospital. Patients identified to have uveitis on assessment were invited to attend a study day at a research eye clinic in Connaught Government Hospital, where a full ophthalmic assessment, fundus photography, ocular coherence tomography were performed and blood tests and chest radiographs were arranged as needed. Funding was provided to patients for transport and food costs associated with study day attendance.

### Inclusion and exclusion criteria

All patients presenting with active or inactive uveitis of any aetiology were invited to participate in the study. Patients were excluded from the study if they were unable to undergo investigations or if they had active viral haemorrhagic fever, such as EVD or Lassa fever.

### Ethical considerations

Study protocols were approved by the Institutional Review Board and Ethics Committee of Ministry of Health and Sanitation, Sierra Leone and Kings College London and adhered to the tenets of the Declaration of Helsinki. Patients who qualified for protocol evaluation were counselled and consented with the assistance of an interpreter in the patient’s Sierra Leone language or dialect (e.g. Krio, Mende, Temne, and others). Patients who were identified to have uveitis were managed according to the medical judgement of the examining physician in partnership with the eye care providers at the specified sites.

### Investigations

Serology testing was conducted in the laboratory of Connaught Government Hospital after training of laboratory personnel and set up of required equipment and reagents, which had been procured and transported from the UK. Validity testing and quality control of tests were executed prior to commencement of the study. Patients in the study assessed to have active uveitis had a panel of blood tests performed and a chest radiograph performed. Blood tests included full blood count, ESR, CRP, syphilis (TPHA), ELISA testing for antibodies (VZV IgM, HSV 1 IgG) and TB QuantiFERON and a HIV test if there were clinical suspicion or if systemic steroids were being started. HIV testing was being provided for free by the HIV service in Sierra Leone. Due to limited reagents, only those with suspected clinical suspicion from history and ocular examination of TB, syphilis, VZV or HSV were offered relevant tests. Other immunological reagents had been procured, including VDRL, VZV IgG, CMV IgG, HSV 1 IgM and HSV2 IgG & IgM, Toxoplasma IgM & IgG, however these failed initial validity testing and therefore were not used.

### Data collection

Data were collected by a team of trained research staff and inputted into an anonymized electronic database. The clinical assessment was performed by ophthalmologists and ophthalmic imaging by a team of trained technicians. Laboratory investigations were performed by trained laboratory technicians. Demographic data including name, age, gender, ethnicity, occupation, medical and ocular history were recorded in the study questionnaire. A general ophthalmic examination included visual acuity testing with a Snellen chart, intraocular pressure measurements and a slit lamp examination including a dilated posterior segment assessment. Cases were classified according to the SUN classification: anatomical type, onset/duration/course, anterior segment flare and cells, vitreous haze and activity [18, 19]. Final aetiological diagnosis was reached through clinical exam, investigations, and clinical impression.

### Retinal imaging

B-scan ultrasonography was performed, when available, for patients without a view of the posterior pole due to media opacity (e.g. cataract, posterior synechiae, vitreous opacity) to assess for vitreous opacity, retinal detachment, or posterior segment pathology. There was a B-scan ultrasound available in the main eye department, so would not always be available to the research eye clinic. Retinal imaging was performed by a trained technician. Macula-centred and mid-peripheral fundus images and macula, disc and retinal nerve fibre layers images were taken using a Zeiss Clarus 700 and a Zeiss Cirrus 6000.

### Outcomes and statistical analysis

SPSS and Microsoft Excel statistical software tools were used. Univariate and multivariate analyses were performed to determine whether demographic variables, clinical presentation (i.e. symptom duration, severity), and anatomic location, and other variables of interest portend a better or poorer visual acuity at presentation and final follow-up. *P*-value < 0.05 was considered statistically significant for all analyses. Visual acuity was measured using Snellen acuity and converted to LogMar for statistical analysis.

## Results

### General characteristics of uveitis patients

132 patients were recruited, 128 of these patients were included as part of the study. 3 patients were excluded due to missing data and 1 was excluded as they had been misdiagnosed as uveitis and instead had a pigmentary retinopathy.

The clinical characteristics of the 128 study patients are shown in Table 1. The median age was 34 (interquartile range=19; range 5 to 74 years). There were 9 children under the age of 18 years old. Males and female study patients occurred in equal numbers. Patients were predominantly from the Western Urban Area (26.6%, *n* = 30), which comprises the capital Freetown, and originated mainly from the Temene (25.6%), Mende (18.8%) and Limba (18.0%) ethnic groups. Most patients originated from rural areas of Sierra Leone (53%), rather than urban (43%). Patients reported their highest educational attainment as tertiary or university level (41%), secondary school (36%) and primary school (21%).

**Table 1:** Summarises demographic and clinical characteristics of recruited study patients.

Characteristics	Patients, *n* (%)
Study patients	
Total recruited	132
Excluded	4
No uveitis	1
Missing data	3
Total included in analysis	128
Demographics	
Median age, years, (IQR; range)	34 (19; 5–74)
Male gender, *n* (%)	63 (50)
Uveitis risk factors	
HIV positive	9 (7)
Past ebola infection	2 (2)
Associated trauma	48 (38)
Clinical classification	
Bilateral	40 (31)
Unilateral	88 (69)
Total eyes affected by uveitis	168
Active	75 (59)
Inactive	53 (41)
Total	128
Ocular clinical findings	
Cataract	51 (30)
Glaucoma	34 (20)
Retinal detachments	23 (14)
Epiretinal membrane	5 (3)
Optic nerve damage	3 (2)
Scleritis	0
Band keratopathy	0
Total eyes	168
Retinal detachments	
Rhegmatogenous	3
Serous	5
Undifferentiated	13
No fundal view, B scan not confirmed	2
Total eyes	23
Unilateral RD	19
Bilateral RD	2
Total	21
Anatomical classification	
Anterior	24 (19)
Intermediate	8 (6)
Posterior	36 (28)
Panuveitis	51(40)
History of uveitis, anatomical location undetermined	9 (7)
Total	128
Laboratory aetiology	
QuantiFERON-gold positive	12 (32)
Total tested	38
VZV IgM positive	5 (6)
Total tested	87
HSV IgG positive	61 (88)
Total tested	69
VDRL/TPHA positive	5 (5)
Total tested	107
Aetiology of uveitis	
Undifferentiated	66 (52)
Toxoplasmosis	46 (36)
Trauma	8 (6)
Syphilis	5 (4)
Ebola	2 (2)
HIV	1 (1)
Total	128
Levels of visual impairment of affected uveitis eyes	
Normal <6/12	78 (46)
Mild – visual acuity worse than 6/12 to 6/18	9 (5)
Moderate – visual acuity worse than 6/18 to 6/60	23 (14)
Severe – visual acuity worse than 6/60 to 3/60	3 (2)
Blindness – visual acuity worse than 3/60	55 (33)
Total	168
Mean Adjusted LogMar VA of all affected eyes, *n*	0.79, 168, 95% CI 0.61, 0.98
Mean LogMar VA of worse affected eye, *n*	1.14, 128

### History of presenting complaint

Eye pain (68%) and eye redness (67%) were the two most common presenting complaints for seeking medical attention. In 30% of patients, the symptoms had been present for over 3 months. The median duration was 30 days and with a range between 1 day to 20 years. Known risk factors for uveitis on history included 9 (7%) HIV positive patients and 2 (2%) Ebola survivors. Forty-eight (38%) patients had a history of associated trauma, although for most of these cases trauma did not appear to be the underlying aetiology of uveitis.

### Uveitis classification

Active uveitis was identified in 75 (59%) of cases. Ocular involvement was unilateral in 88 (68.8%) and bilateral in 40 patients (31.2%), with a total of 168 affected eyes in the study. The most common anatomical type of uveitis was panuveitis (40%), followed by posterior uveitis (28%), anterior uveitis (19%) and intermediate uveitis (6%).

### Ocular findings

Uveitis-associated complications seen in affected eyes included retinal detachment, glaucoma and cataract. Nineteen patients had unilateral or bilateral retinal detachment (14.8%), 34 had glaucoma (26.6%) and 51 had cataract (39.8%). Of the 23 retinal detachments, 3 were rhegmatogenous and 5 were serous. Thirteen cases were classified as undifferentiated, due to their advanced presentation making diagnosis challenging of whether vitreoretinal traction or vitritis occurred first. Two cases had no fundal view and B-scan ultrasonography had not been performed, as ultrasound was unavailable.

We assessed sequelae of uveitis and associated visual impairment. We found that uveitis eyes with retinal detachment had worse vision than those without (adjusted means 1.97 vs 0.70, *p*-value < 0.0001). Those with cataract had worse vision than those without (adjusted means 1.29 vs 0.70, *p* < 0.01). Those with cataract and retinal detachment compared to those without either (adjusted means 1.44 vs 0.45, *p* ≤ 0.00001 had significantly worse vision.

### Aetiology

Most cases were of undifferentiated aetiology (*n* = 66, 52%), followed by toxoplasmosis (*n* = 47, 37%). Trauma was the aetiology of uveitis in 7 patients (5.5%) and syphilis contributed to uveitis in 5 cases (4%). Clinical features of the 5 syphilis uveitis cases included panuveitis, bilateral disease, 360 degrees posterior anterior synechiae, stromal interstitial keratitis, rhegmatogenous or funnel retinal detachment and chorioretinitis.

One patient was considered to have Ebola-related uveitis and another may have been Ebola-related or toxoplasmosis given the clinical assessment. Therefore, up to 2% of cases may have been caused by Ebola-related uveitis. This diagnosis was predominantly made by history and clinical assessment.

107 (83.6%) patients had at least one of the immunological laboratory tests. Of the 58 patients tested, 48% were positive for Quantiferon TB, and 61 patients were positive for HSV IgG (88%). TPHA testing revealed 5 cases (5%) of those tested were positive. VZV IgM was positive in 5 cases (6%) of those tested. None of the quantiferon-positive cases were thought clinically to have TB-uveitis, as there were no clinical signs of granulomatous uveitis in these patients.

## Imaging

**Fig. 1:**
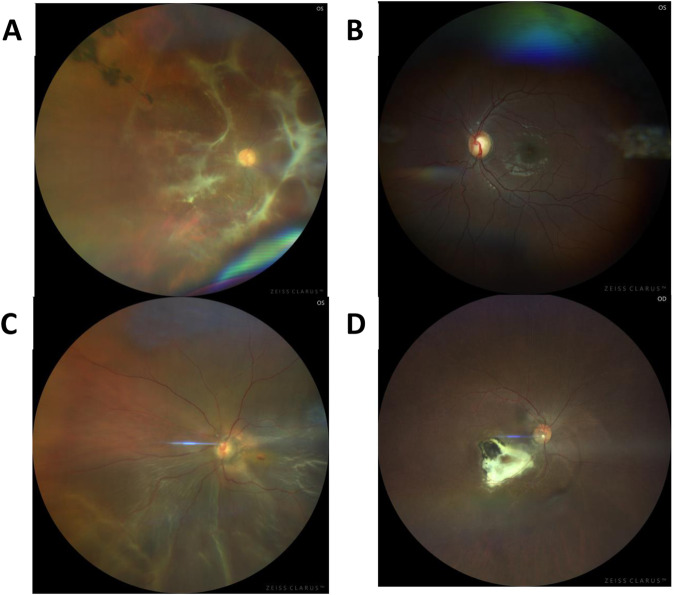
Fundus images were taken with Zeiss Clarus 700 demonstrated ocular sequelae of uveitis. **A** Right fundus photo of chronic vitritis retinal traction. **B** Left fundus photograph with evidence of optic disc cupping, a complication of chronic uveitis. **C** Right fundus photo with inferior retinal detachment and round hole. **D** Right fundus photograph with inactive macula chorioretinal scar.

Fundus photography and OCT assessment helped with clinical assessment of uveitis (Fig. 1).

### Level of blindness

ICD 11 definition of blindness with VA < 3/60 occurred in 33% (*n* = 55) of uveitis affected eyes. The adjusted mean logMar visual acuity of all uveitis affected eyes (*n* = 168) was 0.79, (Snellen equivalent 6/38), and of the uveitis affected eyes and worse affected eyes only in bilateral disease (*n* = 128) the adjusted logMar VA was 1.14, (Snellen equivalent 6/90). Anterior uveitis eyes had significantly better visual acuity than other types, including intermediate, posterior and panuveitis (means of 6/30 vs 6/110, *p* = 0.013).

## Discussion

This is the first study on uveitis in Sierra Leone to explore its epidemiology and aetiology since the EVD epidemic of 2013–16. The primary findings are that approximately one third of individuals with uveitis exhibit bilateral disease and almost 70% have posterior uveitis or panuveitis. Posterior and panuveitis are associated with significantly worse visual acuity than other types of uveitis. Therefore, similar to the Ronday study over 30 years ago, the level and type of uveitis remains associated with significant visual impairment and blindness.

A marker of the severity of uveitis seen is represented in the high prevalence of associated ocular complications including retinal detachment, cataract, and glaucoma. The visual impairment associated with ocular sequelae of uveitis demonstrates the burden of the disease within Sierra Leone. The high level of visual impairment related to uveitis-associated retinal detachments and cataracts compared to even glaucoma, which has been widely reported as causing a significant burden of disease in African countries, demonstrates the need for improved management of uveitis and surgical services to manage these conditions.

Within the cohort of patients examined, up to 2% were found to be associated with Ebola-related uveitis. This suggests that there could potentially be a range of 180 cases to 11,000 cases of uveitis attributed to Ebola based on estimates of Ebola survivors in Sierra Leone. These figures are derived from reported numbers of Ebola survivors and the actual number of individuals affected may be higher. Inferences on aetiology and therefore investigation and treatment of these patients cannot confidently be made based on other countries’ published data. Diagnosis of suspected Ebola uveitis was made in this study predominantly from history and assessment and in the absence of another diagnosis. This can be inferred on the basis that up to 30% of EVD survivors may develop uveitis [20]. One way to confirm the diagnosis in active uveitis or active Ebola infection is through aqueous and vitreous sampling which was not available for this study or in Sierra Leone in general [21]. Post-Ebola aqueous sampling has not identified Ebola from PCR some time following active infection [22]. In both study cases ocular symptoms started following Ebola infection. Ebola uveitis retinal lesions have been previously described [23, 24]. Without utilisation of the fundus photo and OCT images, it is difficult to establish if these retinal lesions are in keeping with these published appearances. Similarly to the limitations described before, EVD survivors are identified by the possession of an Ebola treatment centre discharge certificate. Falsification of these certificates has occurred previously due to the conferred healthcare and other associated benefits.

It is clear from the history that a lot of patients reported prior associated trauma, although this often was not found to be the aetiology of their uveitis. The prevalence of trauma as an aetiology of uveitis varies significantly in the literature, from less than 1 to 12% [25, 26]. Studies have demonstrated that there are higher incidence rates of traumatic injuries in low to middle income compared to high income countries [27, 28]. There is also however significant under-reporting and information registration of cases in these settings, so rates may be significantly underestimated. Traumatic injuries are commonly seen in Sierra Leone due to the type of occupation of participants, such as farmers, fishermen, mechanics and the absence or disuse of protective gear during working hours.

Compared to epidemiological studies on uveitis in the US and US, undifferentiated uveitis and toxoplasmic uveitis make up the largest proportions of uveitis in Sierra Leone. We accept that the ”undifferentiated” cases, were idiopathic in that our set of clinical assessment and investigations may have been unable to pick up the underlying cause, however this is likely because of limited resources and investigations. Undifferentiated uveitis may have been in fact toxoplasmic uveitis, and certainly those patients with active posterior uveitis would be treated as such. It is likely that in countries with a paucity of resources to investigate other differentials, undifferentiated uveitis or toxoplasmic uveitis is over-represented, as they are clinical diagnoses. All active cases of posterior or panuveitis were treated empirically as for Toxoplasmosis, using Co-trimoxazole, Pyrimethamine and initially with Ibuprofen. TPHA, HIV and resting and fasting blood sugars were routinely done for those with Posterior or Pan-uveitis and if normal, then oral prednisolone was initiated later.

Systemic infections causing uveitis, including TB and syphilis are also represented in our cohort. In our cohort, no patients were thought to have uveitis caused by TB as the predominant aetiology, despite 48% of cases tested being QuantiFERON positive. The initial clinical diagnosis did not take into account the results of QuantiFERON, as these were only available once the data collection period had elapsed. TB uveitis diagnosis was predominantly therefore a clinical diagnosis where there was granulomatous uveitis with no other cause. This demonstrates that whilst there is a high prevalence of latent TB in the population generally, few cases within our cohort presented with active TB. Quantiferon testing is often positive in those who are exposed to TB and can be positive in latent or active TB infection. Previous studies have demonstrated that quantiferon TB testing does not have added value in differentiating between latent and active TB in areas of high TB disease burden, and therefore should not be used as a replacement for conventional microbiological diagnosis of TB in these settings [29]. Similarly, our high quantiferon TB results, although being in a cohort of patients with active uveitis, adds to the data that positive testing does not add weight to diagnosis of active TB. None of the positive results led to a change in management of their ocular or systemic condition. However, the benefit of a negative quantiferon result helped with excluding ocular TB as a diagnosis, and to differentiate with other aetiologies, in particular syphilitic uveitis. Additionally, the high positive rates raise the concern that extrapulmonary TB may be more prevalent in Sierra Leone than would previously have been imagined. This small study calls for a more robust research project, in which quantiferon TB testing is systemically performed to look for prevalence of TB in Sierra Leone. Recommendations should include the reliable availability of Quantiferon TB testing in the main laboratory and the routine request for this in cases of uveitis where clinically indicated, such as those with bilateral uveitis, panuveitis or posterior uveitis, in particular to help differentiate from other diagnoses.

Syphilis testing demonstrated 5% of those tested were positive. All these positive syphilis cases had a change of clinical management based on these results, which demonstrates the value of TPHA / VDRL testing in this cohort of patients with uveitis. The diagnosis of Syphilitic Uveitis was made if a positive TPHA test was received and not based solely on clinical findings.

There were challenges with this study. Reasons for reagents failing validity testing included challenges with transportation and storage of reagents and with delays in study commencement. In Sierra Leone there are frequent power outages. Therefore, despite being kept in a fridge whilst in-country, it is likely that they did not remain in 4 degrees Celsius temperature settings, as required. There were also delays with starting the study due to difficulties with procuring necessary laboratory equipment, such as a plate reader. As the study therefore started several months after the reagents had arrived in-country, it is possible that delay may have additionally contributed to them failing validity testing. Additional challenges with laboratory testing included that due to limited supplies and time challenges with procuring further reagents in-country, testing could not be performed for all study patients, and for only those where relevant investigations were more clinically indicated. Finally, limited reagents particularly for quantiferon-TB testing required that collected samples be batch-tested, and therefore results were reported several weeks after patients had been assessed. This meant that investigation results were not immediately useful at the time of the initial clinical diagnosis.

The challenges we faced with validity of reagents and setting up laboratory services in-country, informs us of the need to facilitate a procurement pathway for further supplies of both consumables and equipment, and in particular to ensure a cold-chain pathway for this. There can frequently be procedural challenges in place when transporting large supplies of medical equipment in-country. Close collaboration with the Ministry of Health to facilitate this effectively is crucial. Practical issues of navigating power cuts, such as access to a generator and surge protectors to protect equipment from power surges, are also necessary to plan for when installing valuable resources. Challenges with prompt procurement of consumables meant that serum samples needed to be frozen to be batch-tested when all samples had been collected.

Important limitations to note are that this is a relatively small snapshot study of uveitis in Sierra Leone and that the challenge with reagents and laboratory work posed challenges with aetiological diagnosis of conditions. Furthermore, we do not have the number of patients invited to attend the study day, compared to those who attended. Despite giving study patients an allowance to reduce any financial burden of attending, this may have been a potential source of recruitment bias. Patients were invited to attend from eye clinic settings, which may have also contributed to selection bias.

Recommendations from the findings of this study include that given that a clinical diagnosis of toxoplasmosis was made in 37% of cases, clinicians could treat chorioretinitis or uveitis with no fundal view with antimicrobials, specifically cotrimoxazole in this setting, in conjunction with oral steroids, where non-infectious uveitis is likely. The provision of validated testing for toxoplasmosis serology, syphilis and TB would be invaluable in this setting, and in future work towards using anterior chamber and vitreous samples for PCR testing of fluids to establish aetiologies of uveitis.

This study represents the first attempt to establish local laboratory services within the main governmental hospital in Freetown, aimed at investigating infectious causes of uveitis. As part of this project, laboratory staff have undergone training in-person and remotely on laboratory procedures, handling these specific reagents and equipment and interpreting findings. The utilisation of in-country laboratory facilities presented challenges and limitations, such as power cuts and therefore invalidation of some reagents. However, this project has greater potential of sustainability, increased laboratory expertise and capacity, and continued provision of necessary supplies for improved management of uveitis patients in the future. Laboratory capacity needs to be built on to further characterise infectious and noninfectious causes of uveitis.

The primary findings of this project underscore the continued prevalence of uveitis as a significant cause of visual impairment, particularly in the form of advanced disease as posterior uveitis, panuveitis and bilateral disease. Given the large number of uveitis sufferers in the country and the low number of ophthalmic trained staff available to manage them with limited resources, this condition represents a significant public health concern. Trauma is an important risk factor for uveitis and contributed to a significant proportion of disease. The study highlights substantial advancements in the development and application of laboratory investigations for uveitis diagnosis, yet further efforts are required to enhance the differentiation of its underlying causes. Specifically, it is recommended that additional laboratory studies would aid in identifying the aetiology. Obtaining anterior chamber and vitreous samples for processing would be beneficial in obtaining more definitive diagnoses for infectious aetiologies.

## Summary

### What was known before

- Uveitis causes a significant burden of visual morbidity in Sierra Leone from the last uveitis study in Sierra Leone over 30 years ago.
- Toxoplasmosis associated uveitis caused a significant proportion of uveitis in Sierra Leone.
- The Ebola Virus Epidemic of 2013–16 may have caused a large proportion of uveitis and other ocular complications that has not yet been assessed in Sierra Leone.

### What this study adds

- Ebola associated uveitis contributed to up to 2% of uveitis in this sample of patients with uveitis.
- Uveitis remains a major cause of blindness and visual impairment in Sierra Leone, caused by bilateral disease, posterior and pan-uveitis.
- Syphilis testing in-country in active uveitis patients helped differentiate between other causes of uveitis and affected the management and clinical course of disease for those patients.

## Data Availability

The data that support the findings of this study are not openly available due to reasons of confidentiality and sensitivity and are available from the corresponding author upon reasonable request. Data are located in controlled access data storage at University of Nebraska Medical Centre.
